# The Neutrophil to Lymphocyte Ratio in Poststroke Infection: A Systematic Review and Meta-Analysis

**DOI:** 10.1155/2022/1983455

**Published:** 2022-03-12

**Authors:** Shokoufeh Khanzadeh, Brandon Lucke-Wold, Fatemeh Eshghyar, Katayoun Rezaei, Alec Clark

**Affiliations:** ^1^Student Research Committee, Tabriz University of Medical Sciences, Tabriz, Iran; ^2^University of Florida, Department of Neurosurgery, Gainesville, USA; ^3^Tehran University of Medical Sciences, School of Medicine, Tehran, Iran; ^4^Student Research Committee, University of Kharazmi, Karaj, Iran; ^5^University of Central Florida, School of Medicine, Orlando, USA

## Abstract

Ischemic and hemorrhagic strokes have multiple downstream consequences for patients. One of the most critical is poststroke infection (PSI). The goal of this systematic review and meta-analysis was to critically evaluate the literature regarding the use of the neutrophil to lymphocyte ratio (NLR) as a reliable means to detect early PSI development, particularly poststroke pneumonia (PSP) development to help clinicians institute early interventions and improve outcomes. The following were the inclusion criteria: (1) cross-sectional, case-control, and cohort studies; (2) studies comparing NLR data from PSI or PSP patients to controls; and (3) studies with a control group of stroke patients without infection. There was not any language or publication preference. The Newcastle-Ottawa Scale was used by two writers to assess the quality of the included studies. We assessed the certainty of the associations with GRADE methods. Web of Science, PubMed, and Scopus were searched, and 25 studies were included in the qualitative review. Among them, 15 studies were included in the meta-analysis. Standardized mean difference (SMD) was reported with a 95% confidence interval (CI) for the NLR levels. Patients with PSI had significantly higher NLR levels than stroke patients without infection (SMD = 1.08; CI 95% = 0.78‐1.39, *P* value < 0.001). In addition, the NLR levels of the stroke patients with pneumonia were significantly higher than those without pneumonia (SMD = 0.98; CI 95% = 0.81‐1.14, *P* value < 0.001). However, data extracted from the qualitative review suggested that NLR could not predict urinary tract infection, sepsis, or ventriculitis in stroke patients. Our study indicated that NLR could be recommended as an inexpensive biomarker for predicting infection, particularly pneumonia, in stroke patients. It can help clinicians institute early interventions that can reduce PSI and improve outcomes.

## 1. Introduction

It has been well documented in the cardiovascular literature that the neutrophil to lymphocyte ratio (NLR) is an important marker for clinical outcome [1]. Emerging data from the stroke literature has also highlighted the importance of this ratio as a key marker for outcome [2]. Lux and colleagues found that a high NLR at 24 hours poststroke was associated with poor overall outcomes following ischemic stroke [3]. Kakhki and colleagues similarly linked a high NLR to poor poststroke outcomes [4]. There is a growing body of evidence to support the utility of the NLR as a predictive biomarker for the development of several poststroke complications, including infection, delirium, depression, hemorrhagic transformation, and early neurological deterioration [5, 6]. Of these, infection is both the most common poststroke complication and the best predictor of mortality in stroke patients [7]. Thus, the development of infection is one of the most crucial complications to diagnose early in the poststroke setting. This is critical to detect early as a lot of patients with stroke were previously health and nonsymptomatic. Although poststroke infection (PSI) includes several types of infections, such as urinary tract infection (UTI) and sepsis, poststroke pneumonia (PSP) carries the highest mortality rate in these patients [8]. In recent years, a considerable amount of literature has been published on the predictive role of the NLR in PSI, particularly PSP [4, 9–32]. In fact, the elevated NLR has been postulated to signal an aberrant inflammatory state predisposing to further complications [33]. As studies continue to emerge regarding this important topic, the need for a systematic review to guide clinical decision making is apparent. The key is to understand what an elevated ratio might mean for a patient poststroke to help clinicians institute early interventions and improve outcomes. Some previous studies reported significant association between NLR and several types of PSI, but others did not find any relationship. However, to the best of our knowledge, there was not any systematic review of the available literature performed regarding these important topics. The goal of this systematic review and meta-analysis was to consolidate the available data on the role of the NLR in predicting PSI, particularly PSP, to help guide further clinical management utilizing the predictive capabilities of the NLR.

## 2. Methods

The PRISMA (Preferred Reporting Items for Systematic Reviews and Meta-analysis) guidelines were used to conduct this systematic review and meta-analysis. No registered review protocol exists. Two independent investigators conducted a systematic evaluation of peer-reviewed papers by searching PubMed, Web of Science, and Scopus databases regardless of funding source to find relevant articles published until October 2021. The search was conducted using following keywords: (infection OR bacteraemia OR sepsis OR pneumonia) AND (stroke OR cerebral infarction OR brain infarction OR cerebral hemorrhage OR intracranial hemorrhage) AND (neutrophil-to-lymphocyte ratio OR neutrophil to lymphocyte ratio OR NLR). We did not limit our searching to a specific language or release year. Furthermore, to identify grey literature and further relevant studies, we also conducted a quick nonsystematic search in Google Scholar as a secondary database in English and Chinese, because the majority of identified articles were conducted in China. The Prospero Register was also searched for information on unreleased and ongoing investigations.

### 2.1. Criteria for Inclusion and Exclusion

We identify eligible studies according to the PICOS (population, intervention, control, outcomes, and study design) principle in order to ensure the systematic search of available literature. The inclusion criteria were presented below:

- *Population*: *Patients with PSI*. PSI was defined as sepsis, PSP, UTI, and other types of infection. If a study reported only PSP among stroke patients, it would be excluded from the analysis of differences in NLR level between PSI and controls; then, we would include it in the separate analysis concerning PSP cases solely. This action was taken to increase the homogeneity between studies.
- *Intervention*. NLR
- *Control*. Stroke patients without PSI
- *Outcomes*. The prognostic performance of NLR in PSI and PSP
- *Study Design*. Cross-sectional, case-control, and cohort studies

The criteria for exclusion were as follows: (1) reviews, letters to the editor, animal studies, case series, and case reports; (2) studies using overlapping data; and (3) data in the absence of a control group.

### 2.2. Data Extraction

One investigator extracted the data, which was then double-checked by another. The first author, year of publication, language, study location, study design, age group (adult or children), mean age, male %, total sample size, number of cases and controls individually, and NLR level data in cases and controls were all extracted. A third author was consulted to establish a consensus when there were disputes.

### 2.3. Quality Assessment of Included Studies

The Newcastle-Ottawa Scale (NOS), which includes three sections: selection (4 items), comparability (2 items), and outcome (3 items), and a total score of 0 to 9, was used by two writers to assess the quality of the studies included. Any disagreements were finally resolved through arbitration by a third author.

### 2.4. Statistical Analysis

Standard mean differences (SMDs) were used to accommodate the differences in NLR measurement techniques across various studies. In our study, SMDs with a 95% confidence interval (CI) were used to assess NLR differences between PSI or PSP patients and controls. The mean and SD from the median, range, or IQR were calculated using the methodology provided by Wan et al. [34]. The Cochrane *Q*-test and *I*^2^ index were employed to estimate the between-study heterogeneity. It should be emphasized that for Cochrane's *Q*-test, a *P* value of less than 0.1 was considered statistically significant, and *I*^2^ indexes of 0.75, 0.50, and 0.25, respectively, indicated high, moderate, and low levels of heterogeneity. Also, a random effect model was adopted for meta-analysis of heterogeneous results. Otherwise, we used the fixed-effect model. We performed subgroup analysis according to study design and region to identify the source of heterogeneity. In addition, we assessed publication bias by using the funnel plot and Egger's test, which measures the funnel plot's asymmetry. STATA 12.0 was used to perform statistical analyses of the NLR differences between cases and controls (Stata Corporation, College Station, TX, USA). Except where mentioned, we judged *P* value < 0.05 to be statistically significant.

### 2.5. Certainty of Evidence

The certainty of evidence was determined using the GRADE (Grading of Recommendations Assessment, Development and Evaluation) approach by one author for two outcomes investigated in meta-analysis (PSI and PSP). Finally, the assessments were confirmed by the senior author. According to GRADE, observational studies start at low certainty and may be upgraded for dose–response gradient or for large effect, if suspected biases work against the observed direction of effect, and may be downgraded for publication bias, imprecision, indirectness, inconsistency, and risk of bias.

### 2.6. Role of the Funding Source

This review received no external funding or other support.

## 3. Results

### 3.1. Search and Selection of Literature

**Figure 1:**
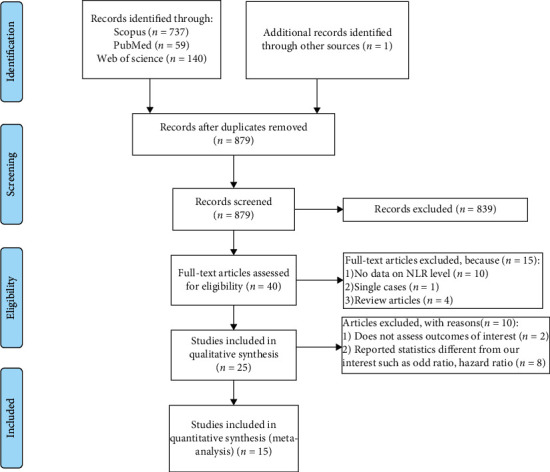
Flow chart of search and study selection.

In this systematic review, the process of discovering and selecting articles is depicted in Figure 1. The first search yielded 59 PubMed records, 140 Web of Science records, and 737 Scopus records. Also, one study was identified through other sources. When 58 duplicate articles were omitted, and a review of the titles and abstracts of the 879 remaining records were conducted, 40 papers were chosen for full-text review. After reading the complete text, 15 of the 40 studies were eliminated due to a lack of data on NLR (*n* = 10), single case (*n* = 1), and review reports (*n* = 4). As a result, the quantitative analysis covered a total of 25 studies [4, 9–32]. Of them, 15 studies comprising 6,410 patients with stroke, 876 of whom developed PSI and 615 of whom developed PSP, were included in the meta-analysis [10, 11, 13, 14, 20, 22, 24–32].

### 3.2. Characteristics of the Included Studies

This systematic review included 25 studies, of which 12 were retrospective [10, 12, 14, 18, 22–25, 28, 30–32] and 13 were prospective [4, 9, 11, 13, 15–17, 19–21, 26, 27, 29]. Among 25 studies, 15 were included in the meta-analysis; nine were retrospective [10, 14, 22, 24, 25, 28, 30–32], and six were prospective [11, 13, 20, 26, 27, 29]. In terms of document language, all of the documents were written in the English language.

Table 1 shows the characteristics of the studies that were included in the meta-analysis, and Table 2 shows characteristics of those that were included only in the qualitative review. In addition, the scale named NOS was used to assess the quality of the included research (Tables 1 and 2). In total, six studies compared NLR levels in PSI patients and controls [11, 20, 22, 24, 27, 31], and nine studies reported NLR levels in the PSP group against controls [10, 13, 14, 25, 26, 28–30, 32].

However, the certainty of this summary estimate of effect was deemed to be very low using the GRADE approach (Table 3).

### 3.3. Difference in NLR Level in Patients with and without PSI

**Figure 2:**
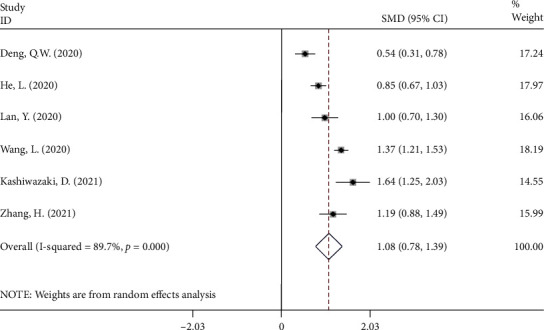
Meta-analysis of differences in NLR level between PSI and NPSI patients.

In 6 cohort studies [11, 20, 22, 24, 27, 31] with 2,416 patients with stroke, NLR levels in the PSI group were compared to those in the nonpoststroke infection (NPSI) group, with 876 patients diagnosed with PSI after the follow-up period. Because the included studies were statistically heterogeneous (*I*^2^ = 89.7%, *P* value < 0.001), the analysis was conducted using the random effect model (Figure 2). The PSI groups had significantly higher NLR levels than the NPSI group (SMD = 1.08; CI 95% = 0.78‐1.39, *P* value < 0.001). However, the certainty of evidence was downgraded to very low for this outcome (Table 3).

**Figure 3:**
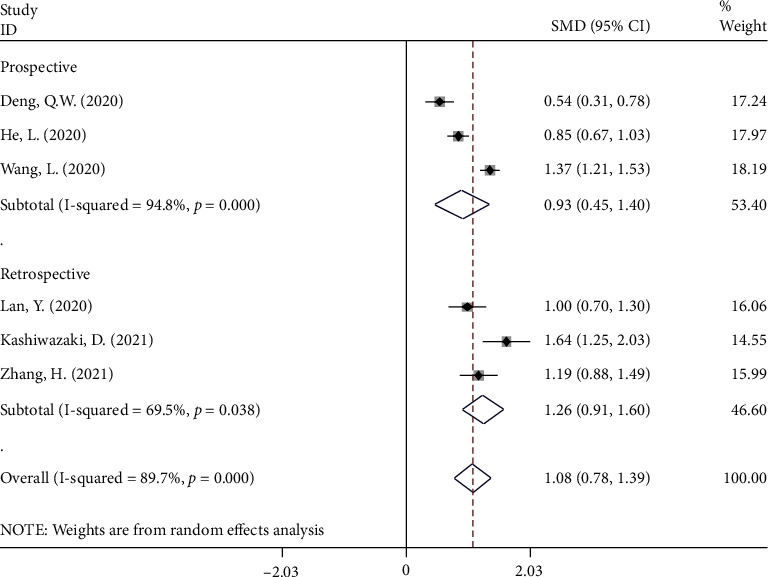
Subgroup analysis of differences in NLR level between PSI and NPSI according to study design.

In the subgroup analysis, according to study design, three were retrospective studies [22, 24, 31] with 779 patients with stroke, 164 of whom developed PSI. Three were prospective studies [11, 20, 27] with 1,637 patients with stroke, 712 of whom developed PSI. In both retrospective and prospective studies, the NLR levels in patients in the PSI group were significantly higher than those in the NPSI group (SMD = 1.26, CI 95% = 0.91‐1.60, *P* value <0 .001 and SMD = 0.93, CI 95% = 0.45‐1.40, *P* value < 0.001, respectively) (Figure 3).

### 3.4. Differences in NLR Level in Patients with and without PSP

**Figure 4:**
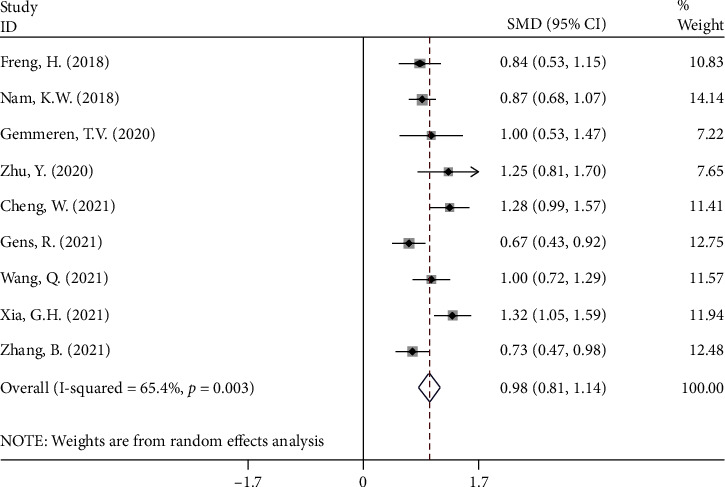
Meta-analysis of differences in NLR level between PSP and NPSP patients.

In 9 studies [10, 13, 14, 25, 26, 28–30, 32] with 3,994 stroke patients, of whom 615 were eventually diagnosed with PSP, NLR levels in the PSP group were compared to those in the nonpoststroke pneumonia (NPSP) group. The NLR levels of the PSP groups were significantly higher than those of the NPSP group (SMD = 0.98; CI 95% = 0.81‐1.14, *P* value < 0.001). Because the included studies were statistically heterogeneous (*I*^2^ = 65.4%, *P* value = 0.003), the meta-analysis was conducted using the random effect model (Figure 4).

**Figure 5:**
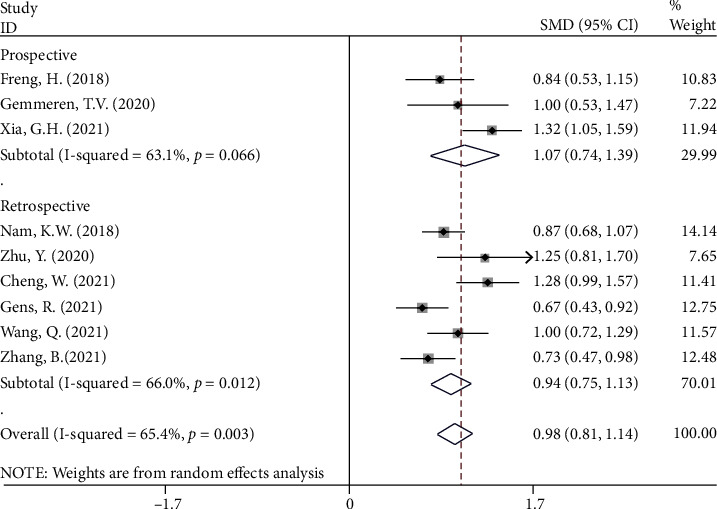
Subgroup analysis of differences in NLR level between PSP and NPSP according to study design.

In subgroup analysis according to study design, there were six retrospective studies [10, 14, 25, 28, 30, 32] with 3,263 patients with stroke, 458 of whom got PSP, and three prospective studies [13, 26, 29] with 731 patients with stroke, 157 of whom got PSP. In both retrospective and prospective studies, the NLR levels in the PSP group were substantially higher than those in the NPSP group (SMD = 0.94, CI 95% = 0.75‐1.13, *P* value < 0.001 and SMD = 1.07, CI 95% = 0.74‐1.39, *P* value < 0.001, respectively) (Figure 5).

**Figure 6:**
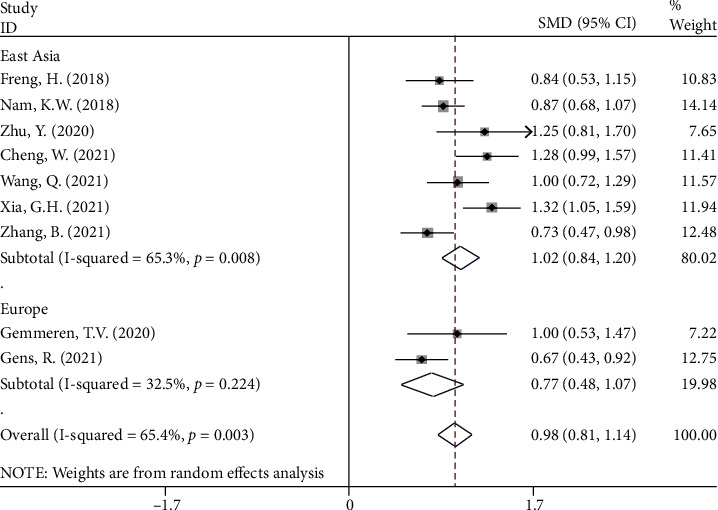
Subgroup analysis of differences in NLR level between PSP and NPSP according to study location.

In another subgroup analysis according to study region, there were seven studies [10, 13, 25, 28–30, 32] in East Asia including 3,385 patients with stroke, of whom 509 had PSP, and two studies [14, 26] in Europe including 609 patients with stroke, of whom 106 developed PSP. In the studies in both East Asia and Europe, the NLR levels in patients with PSP were significantly higher than those in patients with NPSP (SMD = 1.02, CI 95% = 0.84‐1.20, *P* value < 0.001 and SMD = 0.77, CI 95% = 0.48‐1.07, *P* value < 0.001, respectively) (Figure 6).

### 3.5. Publication Bias Assessment

**Figure 7:**
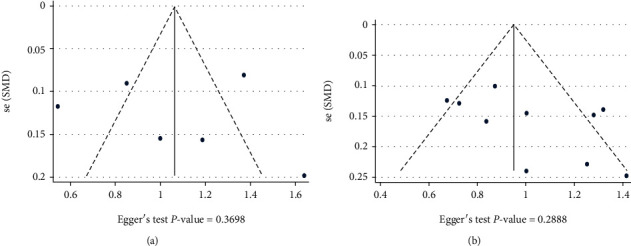
(a) Publication bias assessment based on Funnel plot and Egger's test in data of PSI. (b) Publication bias in data of PSP.

The funnel plot and Egger's test were used to assess the publication bias. As demonstrated in Figure 7, there was no evidence of publication bias in research on the role of NLR in PSI or PSP (Egger's test *P* value = 0.36 and 0.28, respectively).

## 4. Discussion

Our study had two main findings. First, the NLR level was significantly elevated in PSI patients. Second, patients with PSP had a higher level of NLR compared to the NPSP group. It is important to note the dynamic roles of neutrophils and lymphocytes in the setting of a stroke to understand the importance of their relative proportion in the clinical context. It is well-known that levels of neutrophils measured in patients who have strokes are significantly higher than in nonstroke controls [35]. Further, it has been demonstrated that relative levels of neutrophils are predictive of stroke severity, with higher neutrophil counts predictive of more severe strokes [36]. Further work is needed to address role in predicting hemorrhagic conversion [37]. Other groups have found predictive benefit for acute intracerebral hemorrhage [38]. There are several mechanisms by which neutrophil counts may increase in these conditions, but the exact interplay of mechanisms is not currently well established [39]. It is known that neutrophils play a central role in eliminating infarcted neural tissue in the early poststroke period [40]. In the immediate poststroke period, neutrophils migrate to areas of insufficient blood flow via extravasation from nearby blood vessels [41]. Once there, neutrophils target neurovascular units and become activated after the length of ischemia is sufficiently prolonged, releasing mediators such as enzymes through the formation of extracellular traps (NETs) [42]. Pathways involving gene expression appear to be related to the elevation of neutrophil levels as well, including kynurenine pathway upregulation leading to increased tryptophan oxidation and upregulation of arginase 1 [43]. Although the absolute number of neutrophils is increased in stroke patients, their activity related to bacterial killing, like NETosis and oxidative burst, is significantly impaired [44, 45]. This impairment is one of the important mechanisms of stroke-induced immunosuppression and subsequent infection [45].

In addition to neutrophilia, lymphopenia seen in stroke patients exacerbates the elevation in the NLR, adding to its potential diagnostic utility for these patients [46]. Lymphopenia reflects immune depression in this context [47]. There are several mechanisms that may be at play in mediating the development of lymphopenia, but these interactions are similarly not well established. Possible mechanisms at play include a response to physiological stress under the influence of cortisol and a reduction of available regulatory T cells [48]. Additionally, lymphopenia may still be present up to 14 days following a stroke [49]. This, in turn, may make these patients more vulnerable to infections such as pneumonia [50]. This is especially true for patients that have failed recanalization, and the systemic inflammatory response index may be of adjuvant utility [51].

Outlining the relevance of elevated NLR in stroke patients forms the basis for understanding its potential diagnostic and prognostic value in the clinical setting. Recent evidence demonstrates that elevated NLR is associated with poor outcomes in stroke patients, including in-hospital mortality rates [52]. Petrone et al. also found that NLR was significantly higher in stroke patients with poor outcomes compared to those with favorable outcomes, and in fact, asserted that high NLR should be considered a predictor for poor prognosis after stroke [53]. The addition of mean platelet volume might even improve accuracy of the NLR results. Such easily obtainable and not expensive laboratory biomarkers can have a great role in everyday clinical practice and management of stroke patients [54]. Recent studies have demonstrated a variable degree of evidence supporting the diagnostic utility of NLR in predicting PSI rates, particularly rates of PSP development [4, 9–32]. A retrospective study by Nam et al. demonstrated that the predictive potential of NLR further increased when combined with the Pneumonia Severity Index (*P* < 0.001) [25]. In another study, researchers suggested that the NLR by itself was a weak predictor of pneumonia development and was much stronger when used in conjunction with a prediction model that considered other factors such as age, gender, and dysphagia [55]. As such, it appears that although the NLR ratio has demonstrated utility in predicting pneumonia outcomes in stroke patients on its own, using this marker in conjunction with other models and diagnostic tools, such as the Pneumonia Severity Index, may greatly increase its predictive ability [11].

In this context, it appears that prospective and retrospective studies also demonstrate varying degrees of support for the diagnostic utility of the NLR in predicting PSP and other infection rates. In general, retrospective studies currently indicate more support for the diagnostic utility of NLR as a predictor of PSP and other infection development. However, both types of studies have demonstrated statistical significance on this front.

In addition, some studies were included solely in the qualitative review [4, 9, 12, 15–19, 21, 23]. The majority of them assessed the relationship between NLR and PSP [4, 9, 12, 15–18, 21, 23] and reported that NLR could predict pneumonia after stroke, similar to our findings. UTI is another type of PSP whose relationship with NLR was investigated in included studies. Gens et al. showed that NLR within 24 h after stroke onset was not statistically different among patients developing UTI than those without UTI (*P* value = 0.074) [14]. This finding is in agreement with findings reported by Kashiwazaki et al. (*P* value = 0.948) [22], Hou et al. (*P* value = 1) [21], Giede-Jeppe et al. (*P* value = 0.13) [15], and Wang et al. (*P* value = 0.10) [27]. In accordance with these studies, it seems that NLR cannot predict UTI after stroke.

Also, a number of researchers have sought to determine the relationship between NLR and sepsis after stroke. Kashiwazaki et al. found that there was not any association between NLR and sepsis after either ischemic (*P* value = 0.19) or hemorrhagic (*P* value = 0.97) stroke (overall *P* value = 0.34) [22]. Their results agree with the findings of Al-Mufti et al., which showed that admission NLR could not predict the development of sepsis after stroke (*P* value = 0.29) [9]. However, in their study, NLR showed a good predictive value for fever > 101.5°F (*P* value = 0.04) after stroke [9]. In accordance with their results, Giede-Jeppe et al. revealed that NLR could predict the occurrence of neither sepsis nor ventriculitis in patients with stroke due to aneurysmal subarachnoid hemorrhage (*P* value = 0.45; *P* value = 0.87) [17]. These results match those observed in another study by the same researchers on ischemic stroke patients [16]. However, in another study, the same authors revealed that NLR could predict sepsis (*P* value < 0.001) in stroke patients with intracranial hemorrhage [15]. Also, Kim et al. reported that NLR had a predictive role in poststroke sepsis (*P* value < 0.01) [23]. In light of the findings of these studies, we can say that the role of NLR in poststroke sepsis remains controversial.

### 4.1. Strengths and Limitations

This study has several strengths. First, studies were obtained through a systematic search of the literature, augmented with manual searches of reference lists of retrieved papers and systematic reviews. Second, we assessed certainty in the estimates with GRADE, highlighting the remaining uncertainty regarding causal relationships between NLR and PSI or PSP. However, some limitations of our study do exist. Heterogeneity in studies was greater than expected due to various treatment regimens, duration of recorded stays, center protocols, different study populations, different times of blood tests from which NLR was calculated, and different study designs. Therefore, widespread validity is a concern, and future larger prospective studies are needed. Furthermore, several of the studies are limited by bias whether selection or publication, which should be considered. In addition, geographic variability is essential to consider in the context of these results. The majority of current studies on this topic were performed in East Asia and Europe. Given that disparities in both stroke rates and stroke outcomes have been shown between different geological locations, such as Asian and North American countries, it is important to note that the results from the studies on this topic to date may not be as applicable to stroke patients located in different geographical regions [56]. Thus, similar prospective and retrospective studies are warranted in wider geographic locations to characterize any potential differences between these populations. Finally, effect size for several of the tests was limited to a few studies. Thereby widespread adoption and applicability are again a concern warranting further studies. In addition, most studies on the relationship between NLR and PSI have only focused on pneumonia because it is the most common PSI. Researchers have not treated PSI in many details. Searching the databases, we found limited data on the predictive role of admission NLR in sepsis, ventriculitis, and UTI after stroke. Therefore, we could not use the meta-analytic method; however, we reported their findings. Further studies, which take these types of PSI into account, are therefore recommended.

## 5. Conclusion

In conclusion, there has been a recent interest in determining the utility of the NLR as a diagnostic marker for predicting the development of PSP and other infections after stroke. As it currently stands, data collected from prospective and retrospective studies demonstrate a variable degree of support for the predictive potential of the NLR in this context. However, in general, data suggests that the NLR has significant predictive potential for developing PSP and other infections. This predictive potential increases even further when combined with other diagnostic tools such as the Pneumonia Severity Index. Along with administering a larger study to further validate this relationship, additional studies including populations outside of East Asia and Europe are warranted to assess this relationship in a broader context.

## Figures and Tables

**Table 1:** General characteristics of included studies in meta-analysis.

First author	Year of publication	Location	Design	Type of stroke	Mean age	Male (%)	Time of blood test	Time of monitoring presence of infection	NLR in PSI						NLR in PSP						NOS score
									PSI group			NPSI group			PSP group			NPSP group			
									Sample size	Mean	SD	Sample size	Mean	SD	Sample size	Mean	SD	Sample size	Mean	SD	
Freng, H.	2018	East Asia	P	Ischemic	69.32	60.52	Within 24 h of admission	Not declared	—	—	—	—	—	—	50	6.30	2.37	254	4.60	1.96	7
Nam, K.W.	2018	East Asia	R	Ischemic	67.00	60.00	Within 24 h of admission	Within 7 days of admission	—	—	—	—	—	—	112	4.20	3.43	1205	2.58	1.64	8
Deng, Q.W.	2020	East Asia	P	Ischemic	72.33	63.10	Within 24 h of admission	Within 7 days of admission	219	10.16	7.26	114	6.72	3.88	—	—	—	—	—	—	7
Gemmeren, T.V.	2020	Europe	P	Ischemic	74.33	60.00	Within 24 h of admission	Within 7 days of admission	—	—	—	—	—	—	27	6.11	4.77	68	3.48	0.87	9
He, L.	2020	East Asia	P	Ischemic	66.72	48.84	Within 24 h of admission	Within 7 days of admission	194	6.74	2.52	412	4.54	2.62	—	—	—	—	—	—	8
Lan, Y.	2020	East Asia	R	Ischemic	59.34	73.55	Within 24 h of admission	Within 7 days of admission	59	3.99	2.24	198	2.49	1.20	—	—	—	—	—	—	8
Wang, L.	2020	East Asia	P	Ischemic	66.94	48.37	At 36 hours after	Within hospitalization	299	8.65	5.34	499	3.49	2.37	—	—	—	—	—	—	7
Zhu, Y.	2020	East Asia	R	Ischemic	62.00	67.90	Not declared	Within 7 days of admission	—	—	—	—	—	—	31	16.33	13.83	81	6.23	4.22	7
Cheng, W.	2021	East Asia	R	Ischemic	63.94	47.94	Within 24 h of admission	Within 7 days of admission	—	—	—	—	—	—	52	3.83	2.84	682	2.20	1.07	7
Gens, R.	2021	Europe	R	Ischemic	75.03	53.89	Within 24 h of admission	Within 7 days of admission	—	—	—	—	—	—	79	4.10	2.10	435	2.80	1.90	6
Kashiwasaki, D.	2021	East Asia	R	Hemorrhagic	68.10	54.50	Within 8 h of admission	Within hospitalization	54	6.21	1.37	89	4.51	0.77	—	—	—	—	—	—	7
Wang, Q.	2021	East Asia	R	Both types	67.05	73.50	Within 24 h of admission	Within 7 days of admission	—	—	—	—	—	—	64	7.32	4.59	264	3.55	3.53	7
Xia, G.H.	2021	East Asia	P	Ischemic	60.07	70.48	At admission	Within 7 days of admission	—	—	—	—	—	—	80	6.05	4.30	252	2.79	1.49	8
Zhang, H.	2021	East Asia	R	Ischemic	68.00	64.00	Within 24 h of admission	Within 7 days of admission	51	4.28	2.46	328	2.64	1.13	—	—	—	—	—	—	6
Zhang, B.	2021	East Asia	R	Ischemic	63.33	65.50	Within 24 h of admission	Within 7 days of admission	—	—	—	—	—	—	120	10.50	6.90	138	6.53	3.82	6

P: prospective; R: retrospective; NLR: neutrophil to lymphocyte ratio; PSI: poststroke infection; NPSI: nonpoststroke infection; PSP: poststroke pneumonia; NPSP: nonpoststroke pneumonia; SD: standard deviation; h: hours.

**Table 2:** General characteristic of studies included only in qualitative review.

First author	Year of publication	Location	Design	Type of stroke	Mean age	Male (%)	Time of blood test	Time of monitoring presence of infection	Main findings	NOS score
Giede-Jeppe, A.	2017	Europe	P	Hemorrhagic	70.74	46.54	At admission	Within 7 days of admission	The NLR > 4.6 is a good predictor of PSP (*P* value < 0.01) and sepsis (*P* value < 0.01), but not UTI (*P* value = 0.13).	8
Duan, Zh.	2018	East Asia	R	Ischemic	65.66	39.45	Within 4.5 h of admission	Not declared	The NLR > 7.0 is a good predictor of PSP (*P* value < 0.01).	9
Almufti, F.	2019	USA	P	Hemorrhagic	–	31	Within 24 h of admission	Not declared	The NLR ≥ 5.9 is a good predictor of PSP (*P* value < 0.001) and fever (*P* value = 0.02) but not sepsis (*P* value = 0.07).	7
Giede-Jeppe, A.	2019	Europe	P	Hemorrhagic	53	30.73	At admission	Within 7 days of admission	The NLR ≥ 7.05 is a good predictor of PSP (*P* value = 0.01). However, it could not predict either ventriculitis or sepsis (*P* value = 0.87 and *P* value = 0.45, respectively).	7
Giede-Jeppe, A.	2019	Europe	P	Ischemic	72.66	52.8	At admission	Within hospitalization	NLR is independently associated with PSP (risk ratio [95% CI]: 1.083[1.019–1.151] per 1 point increment; *P* = 0.01).	5
Guo, R.	2019	East Asia	R	Hemorrhagic	46.09	57.93	Within 24 h of admission	Within 7 days of admission	The NLR ≥ 8.25 is a good predictor of PSP (*P* value < 0.02).	6
Kakhki, R.D.	2020	Iran	P	Both types	66.96	47.77	At admission	Not declared	The NLR > 5.0 is a good predictor of PSI in patients with ischemic (*P* value = 0.01) stroke but not hemorrhagic (*P* value = 0.11) stroke (overall *P* value < 0.01). Also, it could predict PSP in ischemic (*P* value = 0.03) stroke but not hemorrhagic (*P* value = 0.11) stroke (overall *P* value < 0.01). However, it could not predict UTI and sepsis in either ischemic (*P* value = 0.22 and *P* value = 0.19, respectively) or hemorrhagic (*P* value = 0.1 and *P* value = 0.97, respectively) stroke (*P* value = 0.94 and *P* value =0.34, respectively).	5
Gusdon, A.	2021	USA	P	Hemorrhagic	54	35	Within 5 days of admission	Not declared	The NLR ≥ 8.25 is a good predictor of PSI (*P* value < 0.01).	6
Hou, D.	2021	East Asia	P	Both types	81.29	51.79	At admission	Within hospitalization	The NLR > 5.0 is a good predictor of PSP (*P* value < 0.01) but not UTI (*P* value = 1).	6
Kim, T.J.	2021	East Asia	R	Ischemic	97.7	59.7	At admission	Within hospitalization	The NLR is a good predictor of PSP (*P* value = 0.01) and sepsis (*P* value < 0.01).	5

P: prospective; R: retrospective; NLR: neutrophil to lymphocyte ratio; PSI: poststroke infection; PSP: poststroke pneumonia; UTI: urinary tract infection.

**Table 3:** GRADE evidence profile for cohort studies of the neutrophil to lymphocyte ratio in poststroke infection.

Certainty assessment							No. of patients		Certainty^7^	Importance
No. of studies	Study design	Risk of bias^2^	Inconsistency^3^	Indirectness	Imprecision^5^	Publication bias^6^	Participants, *n*	Cases, *n*		
Poststroke infection										
6	Observational studies	Not serious	Very serious	Not serious	Not serious	None	2416	876	⨁◯◯◯ very low	Critical
Poststroke pneumonia										
9	Observational studies	Not serious	Serious	Not serious	Not serious	None	3994	615	⨁◯◯◯ very low	Critical

^1^Grading of Recommendations Assessment, Development and Evaluation. ^2^Risk of bias based on Newcastle-Ottawa Scale. ^3^When *I*^2^ was <30% inconsistency considered as not serious limitation, >50 considered as serious, and more than 75% considered as very serious limitation. ^5^Serious limitations when there was fewer than 4000 participants for each outcome and very serious limitations when there was fewer than 300 participants for each outcome. ^6^Funnel plot revealed no asymmetry; neither test of publication bias approached *P* < 0.10. ^7^Data from cohort studies begin with a grade of “low.” Downgraded for very serious inconsistency. ^8^Data from cohort studies begin with a grade of “low.” Downgraded for serious inconsistency.

## Data Availability

The datasets generated during and/or analyzed during the current study are available from the corresponding author on reasonable request.
